# Different risks of early-onset and late-onset Parkinson disease in individuals with mental illness

**DOI:** 10.1038/s41531-023-00621-x

**Published:** 2024-01-09

**Authors:** Seo Yeon Yoon, Sang Chul Lee, Jee Hyun Suh, Seung Nam Yang, Kyungdo Han, Yong Wook Kim

**Affiliations:** 1 Department and Research Institute of Rehabilitation Medicine, Yonsei University College of Medicine, Seoul, Republic of Korea; 2 Department of Rehabilitation Medicine, College of Medicine, Ewha Womans University, Seoul, Korea; 3 Department of Physical Medicine & Rehabilitation, Korea University Guro Hospital, Seoul, Republic of Korea; 4 Department of Statistics and Actuarial Science, Soongsil University, Seoul, Republic of Korea

**Keywords:** Parkinson's disease, Risk factors

## Abstract

We aimed to investigate the association of various mental illnesses, including depression, bipolar disorder, schizophrenia, insomnia, and anxiety, with the risk of early-onset Parkinson’s disease (EOPD) (age <50 years) and compare it with that of late-onset PD (LOPD) (age ≥50 years). This nationwide cohort study enrolled 9,920,522 people who underwent a national health screening examination in 2009, and followed up until 31 December 2018. There was a significantly increased risk of EOPD and LOPD in individuals with mental illness, and EOPD showed a stronger association than LOPD (EOPD, hazard ratio (HR) = 3.11, 95% CI: 2.61‒3.72; LOPD, HR = 1.70, 95% CI: 1.66‒1.74; *p* for interaction <0.0001). Our results suggest that people with mental illnesses aged < 50 years are at a higher risk of PD than those aged ≥50 years. Future studies are warranted to elucidate the pathomechanism of EOPD in relation to mental illness.

## Introduction

Although Parkinson’s disease (PD) is a progressive neurodegenerative disease characterized by cardinal motor symptoms, various non-motor symptoms, such as psychiatric problems, sleep disturbance, and autonomic dysfunction, are also present in PD^1^. Psychiatric symptoms are one of the most prevalent non-motor symptoms in PD, and the prevalence of depression and anxiety in PD has been reported to be up to 45% and 56%, respectively^2,3^. They present not only in the advanced stage of PD but also in the prodromal stage, and there have been many reports demonstrating that depression increases PD incidence^4^. Recently, bipolar disorder and schizophrenia have also shown an association with subsequent risk of PD^5,6^.

PD usually occurs in older adults, and its prevalence increases from 0.6% at the age of 65 years to up to 2.6% after the age of 85 years^7^. However, it can develop at much younger ages, and PD with onset before the age of 40 or 50 years is referred to as early-onset PD (EOPD)^8–10^. Disease course of EOPD is different from late-onset PD (LOPD)^10^. EOPD has earlier motor complications, such as dyskinesia and dystonia, and tends to have more psychiatric issues, such as depression, compared to those with LOPD^10,11^. Because individuals with EOPD are diagnosed during the most productive period of their lives, they are likely to retire early, experience social isolation, and have higher psychosocial problems^12,13^.

Although psychiatric problems are common and have a significant impact on the lives of people with EOPD, only a few previous studies have focused on the association between these two conditions^12,13^. It has been suggested that many psychiatric problems are not just symptoms of post-PD, but that various mental illnesses might exist before the diagnosis of PD. Therefore, whether burdensome psychiatric symptoms develop after a diagnosis of EOPD or if individuals with mental illnesses are at an increased risk of EOPD needs to be further investigated. We hypothesized that individuals with mental illness aged <50 years would have a higher risk of PD than those aged ≥50 years. The worldwide incidence of EOPD is significantly increasing; however, it remains almost neglected^11,14^. Therefore, the objective of this study was to investigate the association of various mental illnesses with the risk of EOPD (<50 years) and compare it with that of LOPD (≥50 years) using a nationwide population-based cohort in Korea. We investigated mental illnesses, including depression, bipolar disorder, schizophrenia, insomnia, and anxiety, which have been suggested to be related to PD, for their association with the risk of EOPD. We also analyzed the associations stratified by sex.

## Results

### Characteristics of the participants

Among participants aged <50 years, 330,726 (5.79%) were diagnosed with mental illness. Depression was diagnosed in 89,478 (27.06%), bipolar disorder in 6645 (2.01%), schizophrenia in 10,030 (3.03%), insomnia in 84,293 (25.49%), and anxiety in 201,249 (60.85%). Mean age at the diagnosis of mental illness was 40.14 ± 7.19 years and men constituted 42.95%. People with mental illness were less likely to smoke or drink alcohol and more likely to perform regular physical activity than those without mental illness.

Among participants aged ≥50 years, 738,824 (17.54%) were diagnosed with mental illness. Depression was diagnosed in 230,648 (31.22%), bipolar disorder in 9674 (1.31%), schizophrenia in 8191 (1.11%), insomnia in 239,811 (32.46%), and anxiety in 458,452 (62.05%). Mean age at the diagnosis of mental illness was 63.08 ± 8.79 years and men constituted 33.99%. Table 1 displays the demographic and medical characteristics of participants stratified by age group.

**Table 1:** Characteristics of individuals with mental disorders according to age groups.

	Age <50 years				Age ≥50 years			
	Total (*n* = 5,707,919)	Mental illness			Total (*n* = 4,212,603)	Mental illness		
		No (*n* = 5,377,193)	Yes (*n* = 330,726)	*p* value		No (*n* = 3,473,779)	Yes (*n* = 738,824)	*p* value
Depression	89,478 (1.57)	.	89,478 (27.06)	.	230,648 (5.48)	.	230,648 (31.22)	.
Bipolar disorder	6645 (0.12)	.	6645 (2.01)	.	9674 (0.23)	.	9674 (1.31)	.
Schizophrenia	10,030 (0.18)	.	10,030 (3.03)	.	8191 (0.19)	.	8191 (1.11)	.
Insomnia	84,293 (1.48)	.	84,293 (25.49)	.	239,811 (5.69)	.	239,811 (32.46)	.
Anxiety	201,249 (3.53)	.	201,249 (60.85)	.	458,452 (10.88)	.	458,452 (62.05)	.
Sex, male	3,379,377 (59.21)	3,237,319 (60.2)	142,058 (42.95)	<0.0001	2,037,285 (48.36)	1,786,177 (51.42)	251,108 (33.99)	<0.0001
Age (years)	37.25 ± 7.63	37.07 ± 7.62	40.14 ± 7.19	<0.0001	60.67 ± 8.33	60.16 ± 8.14	63.08 ± 8.79	<0.0001
20 s	1,190,046 (20.85)	1,150,178 (21.39)	39,868 (12.05)	<0.0001				
30 s	1,893,674 (33.18)	1,816,739 (33.79)	76,935 (23.26)					
40 s	2,624,199 (45.97)	2,410,276 (44.82)	213,923 (64.68)					
50 s					2,117,488 (50.27)	1,834,610 (52.81)	282,878 (38.29)	<0.0001
60 s					1,352,803 (32.11)	1,093,254 (31.47)	259,549 (35.13)	
70 s					644,703 (15.3)	476,198 (13.71)	168,505 (22.81)	
80-					97,609 (2.32)	69,717 (2.01)	27,892 (3.78)	
Current smoker	1,849,968 (32.41)	1,771,957 (32.95)	78,011 (23.59)	<0.0001	735,556 (17.46)	647,899 (18.65)	87,657 (11.86)	<0.0001
Heavy drinker	521,124 (9.13)	497,648 (9.25)	23,476 (7.1)	<0.0001	268,711 (6.38)	239,538 (6.9)	29,173 (3.95)	<0.0001
Regular physical activity	887,352 (15.55)	831,968 (15.47)	55,384 (16.75)	<0.0001	893,196 (21.2)	747,286 (21.51)	145,910 (19.75)	<0.0001
Low-income level	1,029,474 (18.04)	958,551 (17.83)	70,923 (21.44)	<0.0001	902,457 (21.42)	753,331 (21.69)	149,126 (20.18)	<0.0001
Metabolic syndrome	861,204 (15.09)	801,848 (14.91)	59,356 (17.95)	<0.0001	1,628,749 (38.66)	1,289,337 (37.12)	339,412 (45.94)	<0.0001
Obesity	1,693,196 (29.66)	1,598,813 (29.73)	94,383 (28.54)	<0.0001	1,543,578 (36.64)	1,272,215 (36.62)	271,363 (36.73)	0.0869
Diabetes mellitus	225,235 (3.95)	208,090 (3.87)	17,145 (5.18)	<0.0001	645,570 (15.32)	516,038 (14.86)	129,532 (17.53)	<0.0001
Hypertension	718,837 (12.59)	662,692 (12.32)	56,145 (16.98)	<0.0001	1,852,589 (43.98)	1,469,852 (42.31)	382,737 (51.8)	<0.0001
Dyslipidemia	611,661 (10.72)	563,581 (10.48)	48,080 (14.54)	<0.0001	1,188,106 (28.2)	926,625 (26.67)	261,481 (35.39)	<0.0001
Body mass index	23.41 ± 3.35	23.42 ± 3.35	23.32 ± 3.35	<0.0001	24.1 ± 3.54	24.1 ± 3.38	24.08 ± 4.2	0.0015
Glucose	93.79 ± 20.25	93.74 ± 20.16	94.69 ± 21.52	<0.0001	102.1 ± 27.4	102.12 ± 27.44	102.04 ± 27.22	0.0386
Cholesterol	190.8 ± 39.2	190.73 ± 39.1	191.94 ± 40.84	<0.0001	201.35 ± 43.6	201.32 ± 43.29	201.5 ± 45.02	0.0009
Systolic blood pressure	119.45 ± 13.73	119.5 ± 13.72	118.57 ± 13.95	<0.0001	126.55 ± 15.83	126.57 ± 15.82	126.44 ± 15.85	<0.0001
Diastolic blood pressure	75.13 ± 9.84	75.16 ± 9.83	74.67 ± 9.93	<0.0001	77.94 ± 10.16	78.04 ± 10.18	77.47 ± 10.03	<0.0001

Values are presented as mean ± SD or number (%).

### EOPD and LOPD risk after mental illness

Among the 5,707,919 participants aged <50 years who were followed up for up to 9 years, 888 (0.2%) people were newly diagnosed with EOPD, of which 144 (0.04%) and 744 (0.01%) were with and without a mental illness, respectively. Among 4,212,603 participants aged ≥50 years, 31,386 (0.75%) individuals were newly diagnosed with LOPD, of which 9765 (1.32%) and 21,621 (0.62%) were with and without a mental illness, respectively. The demographic and clinical characteristics of patients with EOPD and LOPD are shown in Supplementary Table 1.

**Fig. 1:**
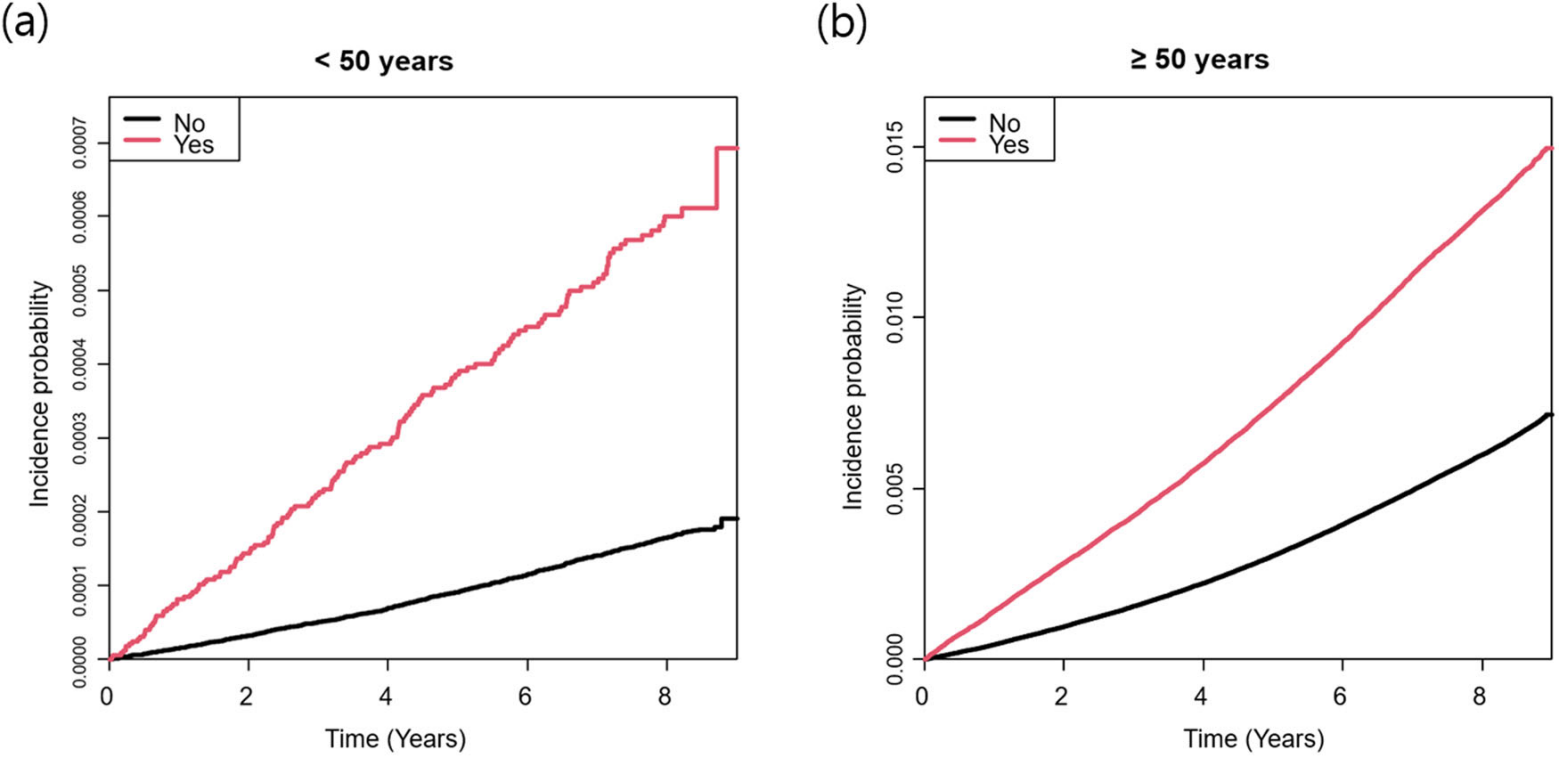
Kaplan‒Meier curves for cumulative incidence of Parkinson’s disease according to age in individuals with mental disorders. The probability of incident Parkinson’s disease in individuals with mental disorders was analyzed according to age. **a** age <50 years and **b** age ≥50 years.

Table 2 presents the HRs for PD development using the univariate and multivariate Cox proportional hazard regression models. After adjusting for confounding variables, there was a significantly increased risk of EOPD and LOPD in individuals with a mental illness. The PD risk in individuals with a mental illness was different across the age groups, showing more significant increase in PD incidence in individuals aged <50 years (age <50 years, hazard ratio (HR) = 3.11, 95% confidence intervals (CI): 2.61‒3.72; age ≥50 years, HR = 1.70, 95% CI: 1.66‒1.74; *p* < 0.0001). When analyzed by sex, people aged <50 years with mental illness had a higher risk of PD than those aged ≥50 years, which was significant for both men and women. For men, the PD risk after mental illness was more than twice as high in individuals aged <50 years than in those aged ≥50 years. Figure 1 displays the results of the Kaplan‒Meier curve analysis and log-rank tests for PD risk in individuals with mental illness according to age group (*p* < 0.0001). In the sensitivity analysis of censoring PD with a combined diagnosis of secondary or atypical parkinsonism (G21-G23), the results were consistent with our main findings (Supplementary Table 2).

**Table 2:** Cox proportional hazard regression analysis on the risk of Parkinson’s disease in individuals with mental disorders stratified by age.

Age (years)	Mental disorders	*N*	PD	Person-years	Incidence rate	Model 1	Model 2	Model 3	Model 4
Total									
Age <50	No	5,377,193	744	36,529,072.7	0.020	1.00	1.00	1.00	1.00
	Yes	330,726	144	1,937,696.7	0.074	3.72 (3.12‒4.45)	3.19 (2.67‒3.81)	3.13 (2.62‒3.74)	3.11 (2.61‒3.72)
Age ≥50	No	3,473,779	21,621	28,153,116.2	0.768	1.00	1.00	1.00	1.00
	Yes	738,824	9765	5,877,001.8	1.662	2.17 (2.11‒2.22)	1.74 (1.70‒1.78)	1.72 (1.68‒1.76)	1.70 (1.66‒1.74)
*p* for interaction						<0.0001	<0.0001	<0.0001	<0.0001
Men									
Age <50	No	3,237,319	477	22,412,088.4	0.021	1.00	1.00	1.00	1.00
	Yes	142,058	86	878,846.4	0.098	4.68 (3.72‒5.89)	3.97 (3.15‒4.99)	3.89 (3.10‒4.90)	3.88 (3.08‒4.88)
Age ≥50	No	1,786,177	11,247	14,275,180.4	0.788	1.00	1.00	1.00	1.00
	Yes	251,108	3553	1,922,247.1	1.848	2.36 (2.27‒2.45)	1.75 (1.68‒1.82)	1.72 (1.65‒1.78)	1.71 (1.64‒1.78)
*p* for interaction						<0.0001	<0.0001	<0.0001	<0.0001
Women									
Age <50	No	2,139,874	267	14,116,984.3	0.019	1.00	1.00	1.00	1.00
	Yes	188,668	58	1,058,850.4	0.055	2.97 (2.23‒3.94)	2.44 (1.84‒3.24)	2.45 (1.84‒3.25)	2.42 (1.83‒3.22)
Age ≥50	No	1,687,602	10,374	1,387,7935.9	0.748	1.00	1.00	1.00	1.00
	Yes	487,716	6212	395,4754.7	1.571	2.10 (2.04‒2.17)	1.73 (1.67‒1.78)	1.72 (1.67‒1.78)	1.69 (1.64‒1.75)
*p* for interaction						0.0184	0.0172	0.0159	0.0134

*PD* Parkinson’s disease.

Incidence rate is the incidence of mortality per 1000 person-years.

Model 1: unadjusted.

Model 2: adjusted for age and sex.

Model 3: adjusted for age, sex, smoking, alcohol consumption, physical activity, income level, and body mass index.

Model 4: adjusted for age, sex, smoking, alcohol consumption, physical activity, income level, body mass index, diabetes mellitus, hypertension, and dyslipidemia.

### EOPD and LOPD risks in people with depression, bipolar disorder, schizophrenia, insomnia, and anxiety

The EOPD and LOPD risks in people with depression, bipolar disorder, schizophrenia, insomnia, and anxiety are presented in Table 3. All mental illnesses evaluated in this analysis were associated with increased risks of EOPD and LOPD. There was a greater increase in the risk of EOPD than LOPD in individuals with depression (*p* < 0.0001), bipolar disorder (*p* = 0.0005), schizophrenia (*p* < 0.0001), insomnia (*p* = 0.0015), and anxiety (*p* = 0.0006). The results of the Kaplan‒Meier curve analysis and log-rank tests for PD risk in individuals with each mental illness according to the age group (*p* < 0.0001) are displayed in Supplementary Figure 1. In the analyses by sex, an increased risk of EOPD compared to that of LOPD in each mental illness was only significant in men. In women, there was no difference in the PD risk after each mental illness between the age groups, except for schizophrenia (age <50 years, HR = 16.85, 95% CI: 9.46‒29.99; age ≥50 years, HR = 5.24, 95% CI: 4.50‒6.11; *p* < 0.0001)

**Table 3:** Cox proportional hazard regression analysis on the risk of Parkinson’s disease according to various mental disorders stratified by age.

			Total	Men	Women
Depression	Age <50	No	1.00	1.00	1.00
		Yes	3.60 (2.70‒4.81)	5.15 (3.60‒7.35)	2.34 (1.44‒3.81)
	Age ≥50	No	1.00	1.00	1.00
		Yes	1.98 (1.91‒2.04)	2.08 (1.97‒2.20)	1.91 (1.83‒2.i00)
	*p* for interaction		<0.0001	<0.0001	0.4219
Bipolar disorder	Age <50	No	1.00	1.00	1.00
		Yes	10.58 (5.99‒18.71)	13.63 (6.78‒27.39)	7.43 (2.77‒19.92)
	Age ≥50	No	1.00	1.00	1.00
		Yes	3.74 (3.33‒4.20)	4.00 (3.35‒4.77)	3.56 (3.05‒4.16)
	*p* for interaction		0.0005	0.0008	0.1484
Schizophrenia	Age <50	No	1.00	1.00	1.00
		Yes	21.20 (15.38‒29.23)	23.91 (16.24‒35.21)	16.85 (9.46‒29.99)
	Age ≥50	No	1.00	1.00	1.00
		Yes	5.29 (4.71‒5.95)	5.33 (4.45‒6.38)	5.24 (4.50‒6.11)
	*p* for interaction		<0.0001	<0.0001	0.0001
Insomnia	Age <50	No	1.00	1.00	1.00
		Yes	2.67 (1.89‒3.76)	3.14 (1.96‒5.02)	2.35 (1.42‒3.89)
	Age ≥50	No	1.00	1.00	1.00
		Yes	1.52 (1.47‒1.58)	1.61 (1.52‒1.70)	1.47 (1.41‒1.54)
	*p* for interaction		0.0015	0.0055	0.0689
Anxiety	Age <50	No	1.00	1.00	1.00
		Yes	2.44 (1.93‒3.10)	2.96 (2.17‒4.04)	2.00 (1.38‒2.90)
	Age ≥50	No	1.00	1.00	1.00
		Yes	1.60 (1.56‒1.65)	1.61 (1.54‒1.69)	1.60 (1.54‒1.66)
	*p* for interaction		0.0006	0.0001	0.2381

Adjusted for age, sex, smoking, alcohol consumption, physical activity, income level, body mass index, diabetes mellitus, hypertension, and dyslipidemia.

The risk of PD after mental illness was classified according to the 10-year-old age group and is presented in Supplementary Figure 2. As age increased, there was a significant decreasing trend in PD risk in individuals with depression (*p* = 0.001), bipolar disorder (*p* < 0.0001), and schizophrenia (*p* < 0.0001).

## Discussion

In this nationwide population-based cohort study, we analyzed 9,920,522 Koreans to evaluate the association between various mental illnesses, including depression, bipolar disorder, schizophrenia, insomnia, and anxiety, and the subsequent risk of EOPD and LOPD. We found that various mental illnesses were associated with the risk of both EOPD and LOPD, and EOPD showed a stronger association with mental illness than LOPD. The risk of EOPD was approximately four times higher in schizophrenia and approximately three times higher in bipolar disorder than in LOPD. In the analyses by sex, an increased risk of EOPD compared to that of LOPD in each mental illness was only significant in men. Our results suggest that people with mental illness aged <50 years are more strongly associated with PD risk than those aged ≥50 years, which is more significant in men.

PD is now considered a non-motor as well as a motor disorder. Among various non-motor symptoms, psychiatric problems, such as depression, anxiety, and sleep disturbance, are common in the course of PD and significantly affect the quality of life (QOL)^15,16^. In recent days, it has been suggested that many psychiatric problems are not just symptoms of PD, but various mental illnesses already exist before its diagnosis. Although it is not yet conclusive whether mental illnesses are prodromal symptoms or risk factors for PD, many previous studies have presented a subsequent risk of PD in various mental illnesses. In a previous study evaluating the association between depression and PD risk, the authors performed sensitivity analyses with 2- or 5-year lag-time of PD diagnosis to consider reverse causality and suggested a consistently increased risk of PD after depression^17^. The risk of PD has been suggested to be increased 2.2 times after depression and 3.3 times after bipolar disorder in previous meta-analyses^4,5^. In a recent study in 2021, the risk of PD increased up to 4.63 times after schizophrenia spectrum disorder^6^. Subjective sleep disturbance, including sleep quality and duration, has been associated with an increased PD risk by ~1.7 times^18^, and women with anxiety have shown double the increased PD risk^19^. Almost all previous studies focused on each mental illness and the risk of PD. In this nationwide study, we included various mental illnesses and evaluated the subsequent PD risk in each disease. Overall, the increased risk of PD after each mental illness in our study was similar to, or higher than, that reported in previous studies. Among various mental illnesses, schizophrenia and bipolar disorder showed the highest risk of developing PD. In this study, we used a claims-based cohort; thus, there could be a possibility of drug-induced Parkinsonism due to the use of antipsychotics in individuals with schizophrenia or bipolar disorder including PD. Nonetheless, our results showed that people aged <50 years with schizophrenia or bipolar disorder have a higher risk of PD or secondary parkinsonism than those aged ≥50 years. Depression, insomnia, and anxiety could be prodromal psychiatric symptoms before PD diagnosis, and it seems possible that EOPD had a higher occurrence of prodromal psychiatric symptoms than LOPD. A sensitivity analysis, censoring PD with a combined diagnosis of secondary or atypical parkinsonism to increase diagnostic accuracy of PD, showed consistent results, which supports a stronger association between mental illness and EOPD than LOPD.

EOPD refers to PD developed at a younger age, before 40 or 50 years, which constitutes 3–10% of all cases of PD^9,20,21^. EOPD has specific clinical features, and it has been suggested that hereditary components play an important role in the origin of EOPD^10,22^. Individuals with EOPD have slower disease progression and preserved cognitive function for a longer duration than those with LOPD^20^. Because of younger age of onset, individuals with EOPD generally live longer with the disease and experience more effect on their lives than with LOPD. These include early retirement, family problems, social isolation, and psychiatric problems, such as depression and anxiety. It seems that psychosocial factors greatly affect the life course of EOPD and contribute to much more impairment of QOL in EOPD than in LOPD^12,13^. However, there have been only a few studies focusing on the association between EOPD and psychiatric conditions^12,13^. The diagnosis of PD is usually based on cardinal motor symptoms; thus, there might be a possibility that prevalent psychiatric symptoms in EOPD could have existed before the diagnosis of PD. In this study, we investigated the association between EOPD and mental illness and found that people with mental illness are at an increased risk of EOPD.

The pathophysiology of PD remains unclear, and both genetic and environmental factors have been suggested to contribute to this phenomenon. EOPD has been suggested to be more related to genetic mutations, such as *SNCA*, *PRKN*, *PINK1*, *PARK7*, and *LRRK*, than LOPD^10^. Among those, *PRKN* mutation has been shown to be related to psychiatric disturbance^23^. However, over 80% of people with EOPD have no familial history or known PD mutations^24^. In this study, we found that various mental illnesses increased the EOPD risk significantly higher than LOPD risk, and this could be added as one of the contributing factors of EOPD origin. The pathophysiology of various mental illnesses associated with PD risk varies from disease to disease. For example, schizophrenia is associated with an overactive dopamine system, which is in the opposite direction to that of PD^25^. A previous study showing a positive association between schizophrenia and PD risk suggested a possible mechanism of increased vulnerability of the dopamine system in the later stages of schizophrenia induced by dopamine dysregulation^6^. As for the link between bipolar disorder and PD, impaired dopaminergic transmission and serotonin have been proposed as possible reasons^26^. In relation to sleep disturbances, it has been reported that sleep deprivation could increase extracellular levels of tau and alpha-synuclein^26^. Monoaminergic neurotransmission and chronic inflammation have been suggested for the association between depression and PD^27,28^. Therefore, future investigations are needed to determine whether a common pathophysiology exists between various mental illnesses and EOPD risk.

In the analyses by sex, an increased risk of EOPD compared to that of LOPD in individuals with mental illness was only significant in men. There have been sex-related differences in the diagnosis, progression, and outcome of PD^29^. Thus, it has been suggested that different pathomechanisms, including environmental, hormonal, and genetic factors, may work depending on the sex of individuals with PD^30^. Our results indicate that individuals with mental illness aged <50 years are at a higher risk of PD than those aged ≥50 years, which is not significant in women. One possible reason is the neuroprotective effect of estrogen in premenopausal females. Estrogen has been shown to have neuroprotective activities against neurotoxins, with anti-inflammatory, anti-apoptotic, and anti-oxidative effects in animal studies^31,32^. Furthermore, estrogen was found to modulate nigrostriatal dopaminergic activity through increased dopamine synthesis and upregulation of neurotrophic factors^33^. Thus, neuroprotective effect of estrogen could offset the high risk of PD in women with mental illness aged <50 years.

This study has several limitations. First, there is a possibility that drug-induced parkinsonism has been included as PD, which might overestimate the risk of PD in our study. Medications used in individuals with various mental illnesses, especially antipsychotics, can cause drug-induced Parkinsonism. In this study, we defined PD using ICD-10 code (G20) and registration code for RIDs (V124). For the registration of RIDs, secondary Parkinsonism should be ruled out, and response to levodopa should also be considered. Nonetheless, the risk of PD was relatively high in individuals with schizophrenia and bipolar disorder, which could be partially related to a misdiagnosis of drug-induced PD. Second, although we tried to include many variables associated with PD development, dietary habits, such as dietary food or caffeine consumption, were unavailable and not included in the analysis. Third, we could not elucidate the physiological link between mental illness and EOPD in this nationwide cohort study. Future laboratory studies on the pathomechanism of EOPD in relation to mental illness are warranted.

In conclusion, in the analysis of 9,920,522 Koreans, we found that various mental illnesses were associated with the subsequent risk of both EOPD and LOPD, and EOPD showed a stronger association with mental illness than observed LOPD. People aged <50 years with schizophrenia or bipolar disorder had a higher risk of PD or secondary parkinsonism than those aged ≥50 years. And there also be a possibility that EOPD has a higher occurrence of prodromal psychiatric symptoms than LOPD. In the analyses by sex, an increased risk of EOPD compared to that of LOPD in each mental illness was only significant in men. Our results suggest that men with mental illnesses aged <50 years are at a higher risk of PD than those aged ≥50 years, which requires clinical attention. Future studies are warranted to elucidate the pathomechanism of EOPD in relation to mental illness.

## Methods

### Data source

We used data from the Korean National Health Insurance Service (NHIS) and National Health Screening databases for the analysis. The Korean NHIS is mandatory for Korean citizens with ~50 million subscribers. The database contains a unique anonymous number for each individual and includes demographic information as well as information related to medical records and costs, such as diagnoses using the International Classification of Diseases (ICD-10), examinations, prescriptions, and procedures. The NHIS provides a free national health screening program (NHSP) at least every 2 years for all beneficiaries aged ≥40 years and workplace subscribers of all ages. In addition to the physical measurements and laboratory tests, a self-reported questionnaire is administered.

### Study population

**Fig. 2:**
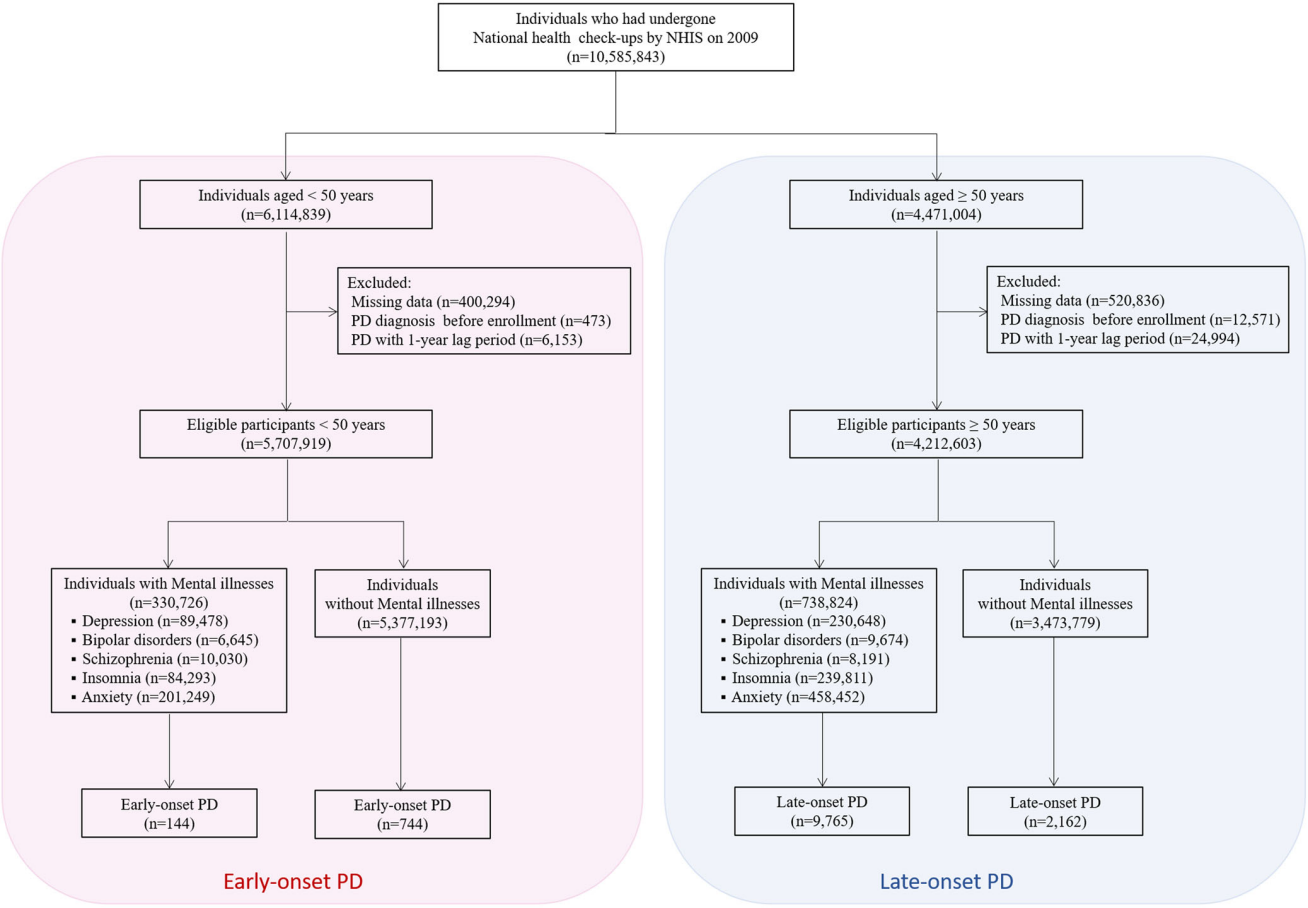
Flowchart of the study population. KNHIS Korean National Health Insurance Service, PD Parkinson’s disease.

Individuals aged ≥20 years, who had undergone a health examination provided by the NHIS in 2009, were enrolled. After excluding individuals with missing data, those who had a diagnosis of PD before enrollment were also excluded. We set a 1-year lag period for PD diagnosis to avoid the risk of reverse causality, and 9,920,544 individuals remained for the analysis. We categorized the remaining participants into two age-specific groups at enrollment and tracked them until 31 December 2018. Finally, 5,707,919 people aged <50 years were enrolled, and participants were censored when they reached the age of 50 years. A total of 4,212,063 individuals aged ≥50 years were included in the study. Among the participants, people with diagnoses of mental illness, including depression (F22, F23), bipolar disorder (F30, F31), schizophrenia (F20), insomnia (F510, G470), and anxiety (F40, F41), were identified based on ICD-10 codes (Fig. 2). This study was approved by the Institutional Review Board of the Korea University Guro Hospital, which waived the requirement for obtaining informed consent from patients.

### Study outcome: Parkinson’s disease

The outcome variable in this study was PD incidence. Since 2001, the South Korean government has operated a registration program for rare intractable diseases (RIDs), including PD, to assist patients with medical expenses. The diagnostic criteria for the PD registration code (V124) for RIDs are almost identical to the UK Parkinson’s Disease Society Brain Bank diagnostic criteria^34^. Newly diagnosed PD between January 2010 and December 2018 was defined based on the ICD-10 code (G20) and a registration code (V124) for PD in the RID program.

### Other variables

Lifestyle factors were assessed using a self-reported questionnaire. Alcohol consumption was classified according to the amount of alcohol consumed per day, and those with alcohol intake per day >30 g were characterized as heavy drinkers. Regular physical activity was defined as engaging in ≥20 min of vigorous-intensity physical activity ≥3 days a week or ≥30 min of moderate-intensity physical activity ≥5 days a week. Individuals who paid the bottom 20% of NHI premiums were characterized to be in the low-income group. Anthropometric data, such as height and weight, were measured, and body mass index (BMI) was calculated as weight divided by height squared (kg/m^2^). Obesity was defined as BMI >25. Laboratory measurements, such as fasting plasma glucose and total cholesterol levels, were conducted after overnight fasting. The baseline comorbidities of individuals were identified based on a combination of medical history information, ICD-10 codes, laboratory data, and prescribed medications.

### Statistical analyses

Differences in participant characteristics were assessed using an independent *t* test for continuous variables or chi-squared test for categorical variables. Continuous variables are expressed as mean ± standard deviation, while categorical variables are presented as numbers (percentages). The crude incidence rate (IR) of new-onset PD was calculated as the number of events per 1000 person-years (PYs). Univariate and multivariate Cox proportional hazards regression models were used to evaluate the risk of PD according to the presence of mental illness. We used four progressive models: Model 1 was unadjusted; Model 2 was adjusted for age and sex; Model 3 was further adjusted for smoking, alcohol consumption, physical activity, income level, and BMI; and Model 4 was further adjusted for diabetes mellitus, hypertension, and dyslipidemia. We investigated the risk of EOPD (<50 years) and LOPD (≥50 years) after mental illness and evaluated the differences between the two age groups. The risks for PD are presented as HRs and 95% CIs. We also performed a sensitivity analysis censoring PD with a combined diagnosis of secondary parkinsonism or atypical parkinsonism (G21-G23) to increase diagnostic accuracy of PD. The cumulative incidence of PD according to mental illness was calculated using Kaplan‒Meier curves and the log-rank test. A *p* value < 0.05 was considered statistically significant. Data were analyzed using the SAS version 9.4 software (SAS Institute, Cary, NC, USA).

### Reporting summary

Further information on research design is available in the Nature Research Reporting Summary linked to this article.

### Supplementary information

Supplementary materials

Reporting summary

## Data Availability

The source NHIS data do not belong to the researchers and we are not allowed to transfer data file to a third party under Korean law. The data can be used after approval of the Institutional Review Board and the Korea NHIS Big Data Operations Department (https://nhiss.nhis.or.kr/bd/ay/bdaya 001 iv.do).

## Code availability

The code used for the current study is available from the corresponding authors on reasonable request.
