# Supplementary material for: Different risks of early-onset and late-onset Parkinson disease in individuals with mental illness
Source: NPJ Parkinsons Dis. 2024 Jan 9;10:17. doi: 10.1038/s41531-023-00621-x (PMC10776668; doi:10.1038/s41531-023-00621-x)
Supplement: Supplementary file 1 — Supplementary materials [file 41531_2023_621_MOESM1_ESM.pdf]

Supplementary Table 1. Characteristics of individuals with early-onset and late-onset Parkinson's disease

	Early-onset Parkinson's disease (<50 years)			Late-onset Parkinson's disease (≥50 years)		
	No (n=5,707,031)	Yes (n=888)	*p*-value	No (n=4,181,217)	Yes (n=31,386)	*p*-value
Mental disorders	330,582 (5.79)	144 (16.22)	<.0001	729,059 (17.44)	9,765 (31.11)	<.0001
Depression	89,429 (1.57)	49 (5.52)	<.0001	226,782 (5.42)	3,866 (12.32)	<.0001
Bipolar disorder	6,633 (0.12)	12 (1.35)	<.0001	9,389 (0.22)	285 (0.91)	<.0001
Schizophrenia	9,991 (0.18)	39 (4.39)	<.0001	7,907 (0.19)	284 (0.9)	<.0001
Insomnia	84,259 (1.48)	34 (3.83)	<.0001	236,427 (5.65)	3,384 (10.78)	<.0001
Anxiety	201,175 (3.53)	74 (8.33)	<.0001	452,340 (10.82)	6,112 (19.47)	<.0001
Sex, Male	3,378,814 (59.2)	563 (63.4)	0.0109	2,022,485 (48.37)	14,800 (47.15)	<.0001
Age	37.25±7.63	40.52±4.72	<.0001	60.63±8.32	67.03±7.66	<.0001
20s	1,190,017 (20.85)	29 (3.27)				
30s	1,893,441 (33.18)	233 (26.24)	<.0001			
40s	2,623,573 (45.97)	626 (70.5)				
50s				2,112,011 (50.51)	5,477 (17.45)	
60s				1,340,251 (32.05)	12,552 (39.99)	<.0001
70s				632,640 (15.13)	12,063 (38.43)	
80-				96,315 (2.3)	1,294 (4.12)	
Current smoker	1,849,728 (32.41)	240 (27.03)	0.0006	732,287 (17.51)	3,269 (10.42)	<.0001
Heavy drinker	521,040 (9.13)	84 (9.46)	0.7331	267,321 (6.39)	1,390 (4.43)	<.0001
Regular physical activity	887,212 (15.55)	140 (15.77)	0.8566	886,884 (21.21)	6,312 (20.11)	<.0001
Low income level	1,029,278 (18.04)	196 (22.07)	0.0018	896,489 (21.44)	5,968 (19.01)	<.0001
Metabolic syndrome	861,041 (15.09)	163 (18.36)	0.0065	1,613,801 (38.6)	14,948 (47.63)	<.0001
Obesity	1,692,905 (29.66)	291 (32.77)	0.0427	1,531,671 (36.63)	11,907 (37.94)	<.0001
Diabetes mellitus	225,188 (3.95)	47 (5.29)	0.0392	638,705 (15.28)	6,865 (21.87)	<.0001
Hypertension	718,698 (12.59)	139 (15.65)	0.006	1,835,414 (43.9)	17,175 (54.72)	<.0001

Dyslipidemia	611,522 (10.72)	139 (15.65)	<.0001	1,178,037 (28.17)	10,069 (32.08)	<.0001
Body mass index	23.41±3.35	23.75±3.44	0.0027	24.09±3.54	24.16±3.01	0.0007
Glucose	93.79±20.25	95.5±18.46	0.0119	102.08±27.38	104.89±30.21	<.0001
Cholesterol	190.8±39.2	194.64±36.43	0.0035	201.37±43.56	197.93±47.77	<.0001
Systolic blood pressure	119.45±13.73	120.09±13.96	0.1635	126.53±15.82	128.1±16.08	<.0001
Diastolic blood pressure	75.13±9.84	75.77±9.8	0.0514	77.94±10.16	77.8±10.17	0.0146

Values are presented as mean ± SD or number (%)

Supplementary Table 2. Cox proportional hazard regression analysis on the risk of Parkinson's disease in individuals with mental disorders when censoring Parkinson's disease with a combined diagnosis of secondary or atypical parkinsonism

Age (years)	Mental disorders	N	PD	Person-years	Incidence rate	Model 1	Model 2	Model 3	Model 4
**Total**									
Age < 50	No	5,377,193	696	36,529,072.7	0.019	1.00	1.00	1.00	1.00
	Yes	330,726	142	1,937,696.7	0.073	3.92 (3.27–4.70)	3.35 (2.80–4.02)	3.29 (2.74–3.94)	3.27 (2.73–3.92)
Age ≥ 50	No	3,473,779	20,710	28,153,116.2	0.736	1.00	1.00	1.00	1.00
	Yes	738,824	9,428	5,877,001.8	1.604	2.18 (2.13–2.24)	1.75 (1.70–1.79)	1.73 (1.69–1.77)	1.71 (1.67–1.75)
p for interaction						<.0001	<.0001	<.0001	<.0001
**Men**									
Age < 50	No	3,237,319	447	22,412,088.4	0.020	1.00	1.00	1.00	1.00
	Yes	142,058	84	878,846.4	0.096	4.87 (3.86–6.15)	4.13 (3.27–5.21)	4.05 (3.21–5.12)	4.04 (3.20–5.10)
Age ≥ 50	No	1,786,177	10,771	14,275,180.4	0.755	1.00	1.00	1.00	1.00
	Yes	251,108	3,426	1,922,247.1	1.782	2.37 (2.83–2.474)	1.76 (1.69–1.83)	1.72 (1.66–1.79)	1.72 (1.65–1.79)
p for interaction						<.0001	<.0001	<.0001	<.0001
**Women**									
Age < 50	No	2,139,874	249	14,116,984.3	0.018	1.00	1.00	1.00	1.00
	Yes	188,668	58	1,058,850.4	0.055	3.18 (2.39–4.236)	2.61 (1.96–3.47)	2.61 (1.96–3.48)	2.60 (1.95–3.46)
Age ≥ 50	No	1,687,602	9,939	13,877,935.9	0.716	1.00	1.00	1.00	1.00
	Yes	487,716	6,002	3,954,754.7	1.518	2.12 (2.05–2.19)	1.74 (1.68–1.79)	1.73 (1.68–1.79)	1.70 (1.65–1.76)
p for interaction						0.0059	0.0055	0.005	0.0039

PD, Parkinson's disease
Incidence rate is the incidence of mortality per 1000 person-years.
Model 1: unadjusted
Model 2: adjusted for age and sex
Model 3: adjusted for age, sex, smoking, alcohol consumption, physical activity, income level, and body mass index
Model 4: adjusted for age, sex, smoking, alcohol consumption, physical activity, income level, body mass index, diabetes mellitus, hypertension, and

dyslipidemia

Supplementary Figure 1. Kaplan-Meier curves for cumulative incidence of Parkinson disease according to age in individuals with (a) depression, (b) bipolar disorder, (c) schizophrenia, (d) insomnia, and (e) anxiety. The probability of incident Parkinson's disease according to age in individuals with mental disorders was analyzed using Cox proportional hazard regression analysis.

(a)

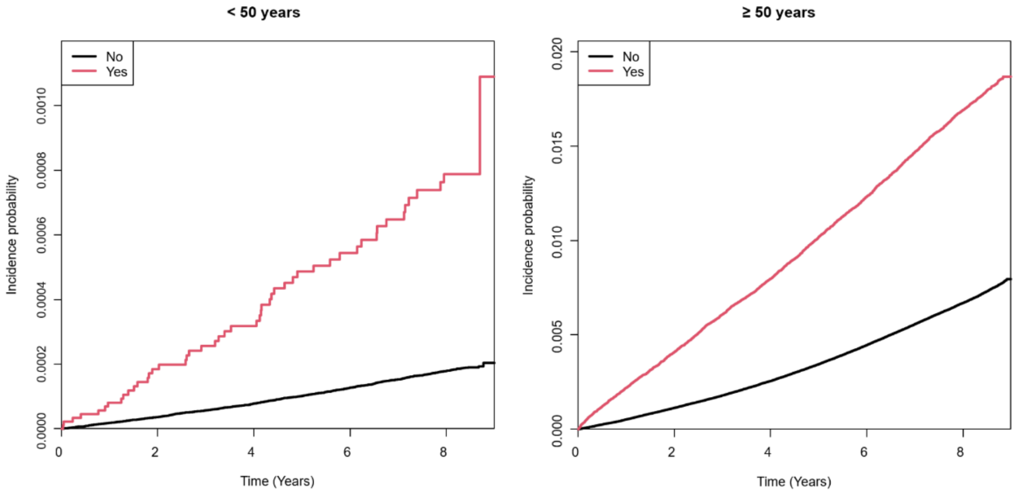

(b)

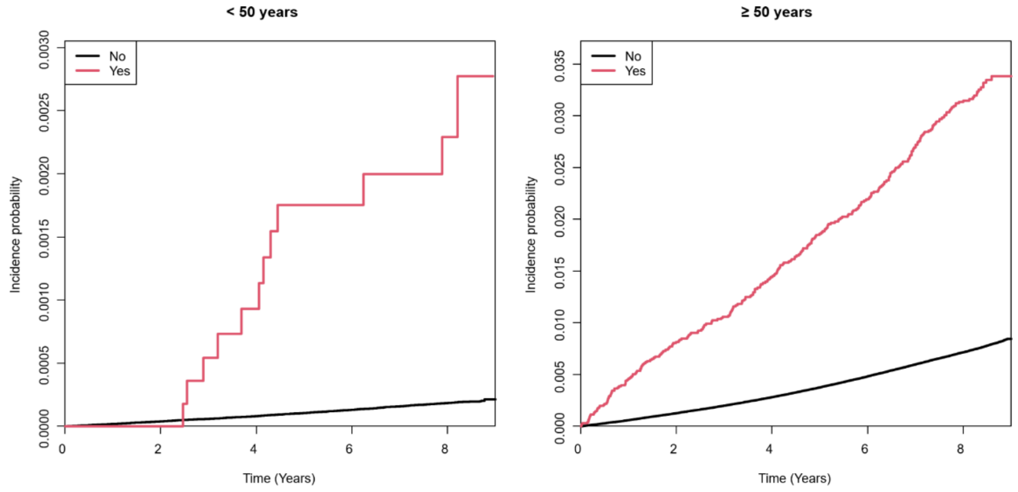

(c)

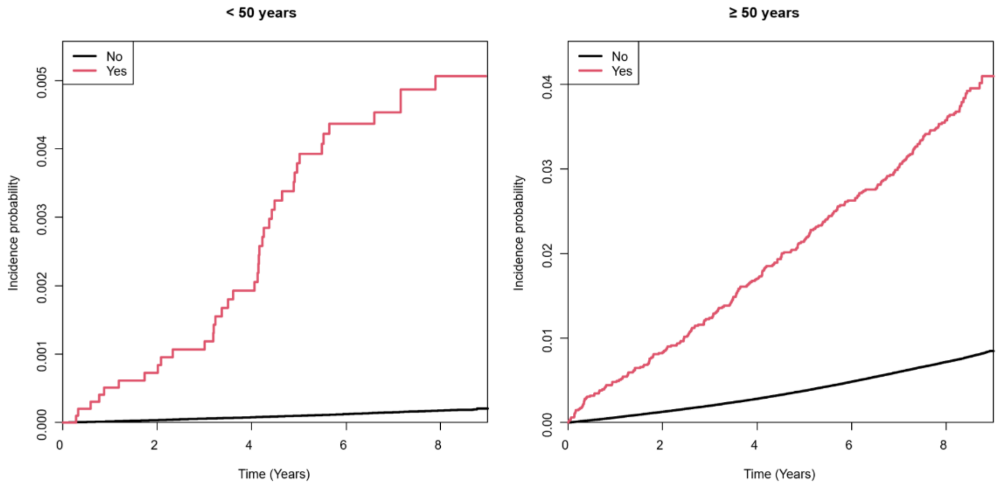
< 50 years
≥ 50 years
No
Yes
Incidence probability
Time (Years)
0.000
0.001
0.002
0.003
0.004
0.005
0.00
0.01
0.02
0.03
0.04
0
2
4
6
8

(d)

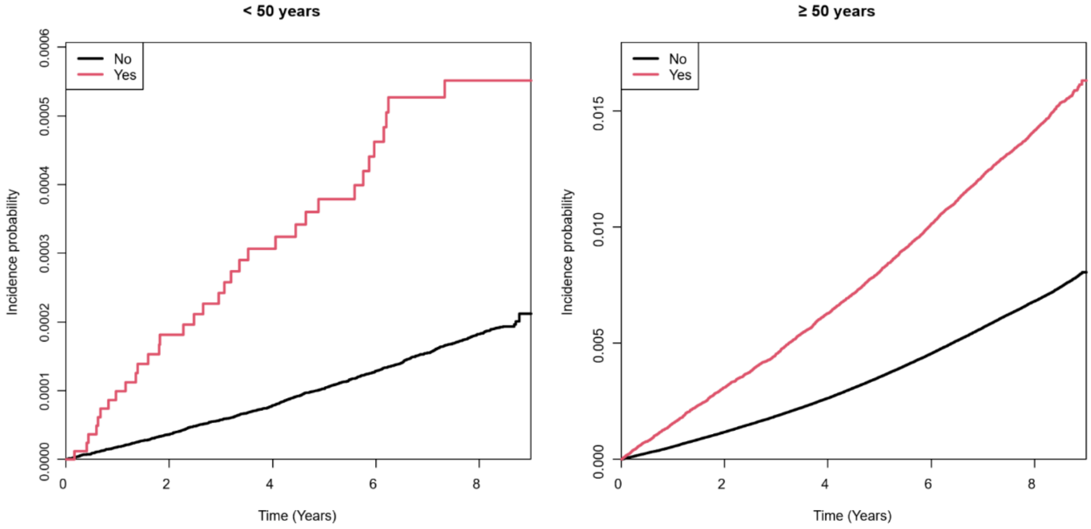
< 50 years
≥ 50 years
No
Yes
Incidence probability
Time (Years)
0.0000
0.0001
0.0002
0.0003
0.0004
0.0005
0.0006
0.000
0.005
0.010
0.015
0
2
4
6
8

(e)

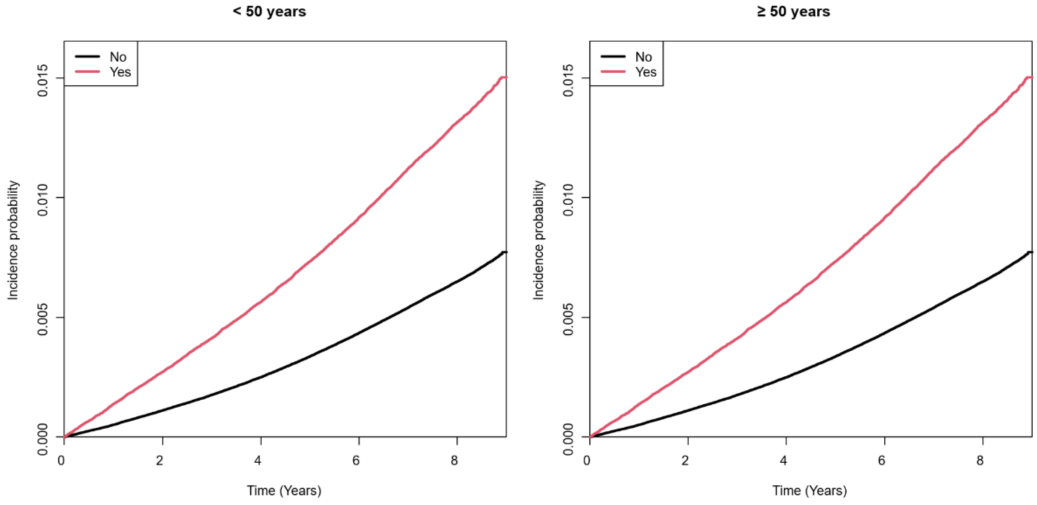
< 50 years
≥ 50 years
No
Yes
Incidence probability
Time (Years)
0.000
0.005
0.010
0.015
0
2
4
6
8

Supplementary Figure 2. Risk of Parkinson's disease stratified by age groups in individuals with (a) mental disorders, (b) depression, (c) bipolar disorders, (d) schizophrenia, (e) insomnia, and (f) anxiety. Adjusted hazard ratios and 95% confidence intervals of Parkinson's disease by age groups. Adjusted for age, sex, smoking, alcohol consumption, physical activity, income level, body mass index, diabetes mellitus, hypertension, and dyslipidemia. Error bars indicate 95% confidence intervals.

(a)

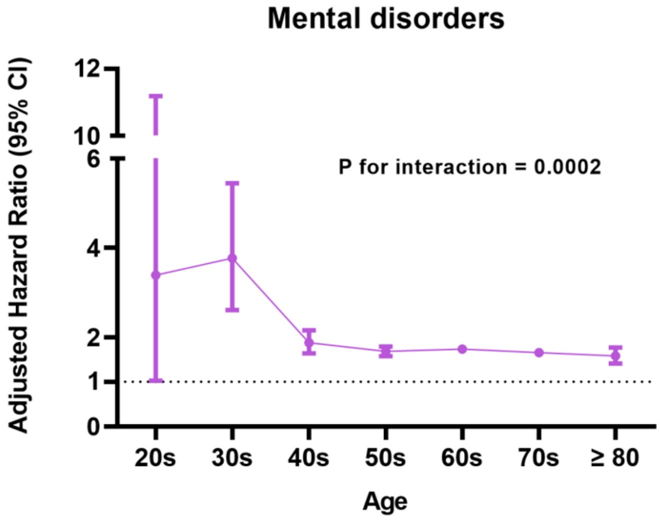

(b)

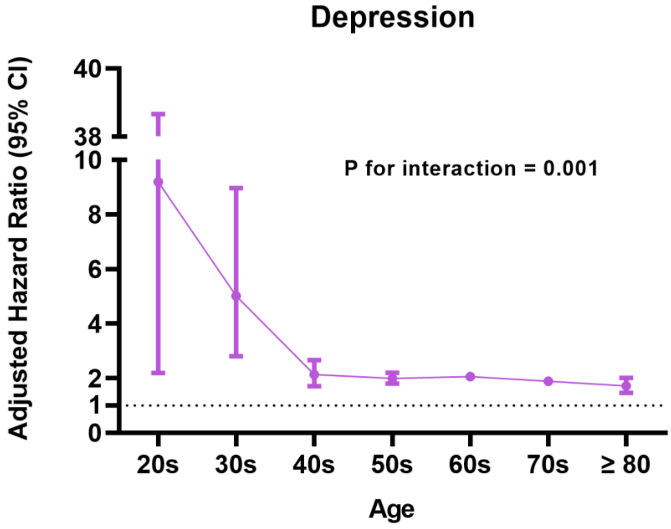

(c)

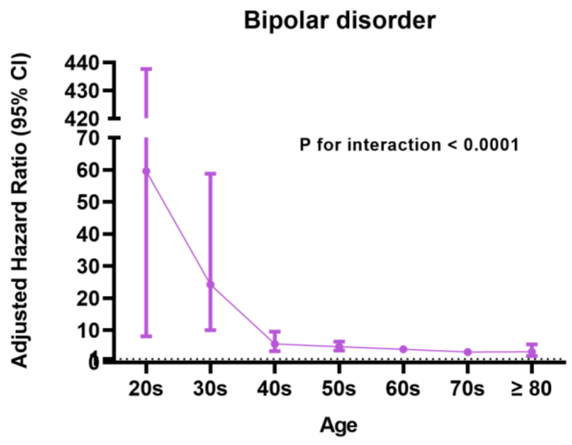
Bipolar disorder
Adjusted Hazard Ratio (95% CI)
440
430
420
70
60
50
40
30
20
10
0
1
P for interaction < 0.0001
20s
30s
40s
50s
60s
70s
≥ 80
Age

(d)

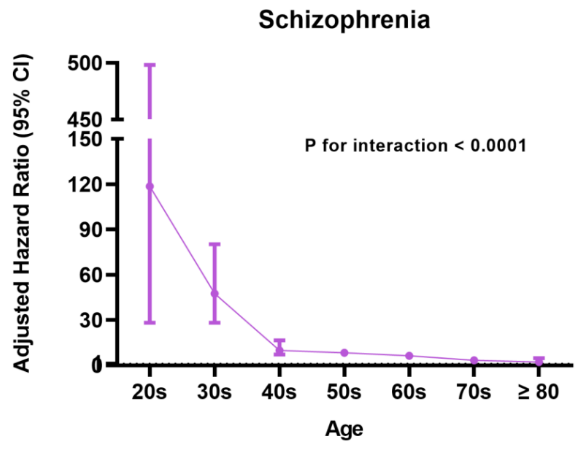
Schizophrenia
Adjusted Hazard Ratio (95% CI)
500
450
150
120
90
60
30
0
1
P for interaction < 0.0001
20s
30s
40s
50s
60s
70s
≥ 80
Age

(e)

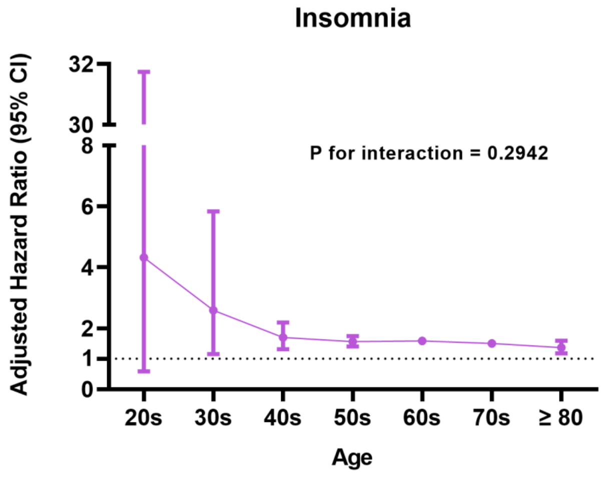
Insomnia
Adjusted Hazard Ratio (95% CI)
32
30
8
6
4
2
1
0
P for interaction = 0.2942
20s
30s
40s
50s
60s
70s
≥ 80
Age

(f)

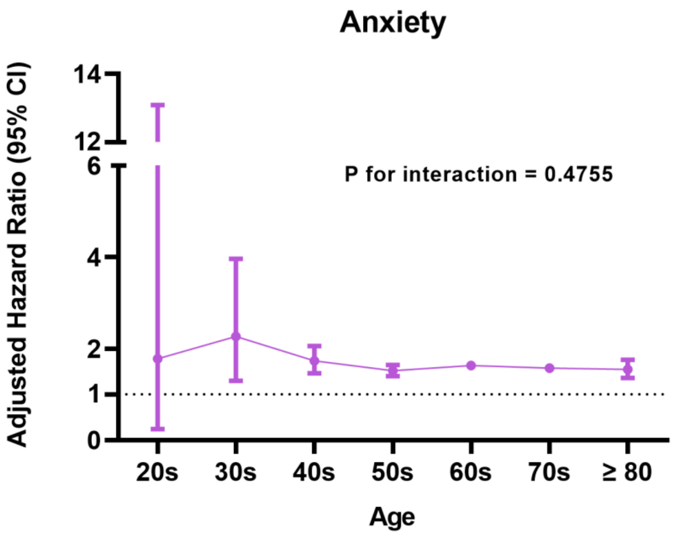
Anxiety
Adjusted Hazard Ratio (95% CI)
14
12
6
4
2
1
0
P for interaction = 0.4755
20s
30s
40s
50s
60s
70s
≥ 80
Age
