# Supplementary material for: Different risks of early-onset and late-onset Parkinson disease in individuals with mental illness
Source: NPJ Parkinsons Dis. 2024 Jan 9;10:17. doi: 10.1038/s41531-023-00621-x (PMC10776668; doi:10.1038/s41531-023-00621-x)
Supplement: Supplementary file 2 — Reporting summary [file 41531_2023_621_MOESM2_ESM.pdf]

nature portfolio

Corresponding author(s): Kyungdo Han
Yong Wook Kim

Last updated by author(s): Nov 7, 2023

Reporting Summary

Nature Portfolio wishes to improve the reproducibility of the work that we publish. This form provides structure for consistency and transparency in reporting. For further information on Nature Portfolio policies, see our Editorial Policies and the Editorial Policy Checklist.

Statistics

For all statistical analyses, confirm that the following items are present in the figure legend, table legend, main text, or Methods section.

n/a	Confirmed	
☐	☒	The exact sample size (*n*) for each experimental group/condition, given as a discrete number and unit of measurement
☐	☒	A statement on whether measurements were taken from distinct samples or whether the same sample was measured repeatedly
☐	☒	The statistical test(s) used AND whether they are one- or two-sided<br>*Only common tests should be described solely by name; describe more complex techniques in the Methods section.*
☐	☒	A description of all covariates tested
☐	☒	A description of any assumptions or corrections, such as tests of normality and adjustment for multiple comparisons
☐	☒	A full description of the statistical parameters including central tendency (e.g. means) or other basic estimates (e.g. regression coefficient) AND variation (e.g. standard deviation) or associated estimates of uncertainty (e.g. confidence intervals)
☐	☒	For null hypothesis testing, the test statistic (e.g. *F*, *t*, *r*) with confidence intervals, effect sizes, degrees of freedom and *P* value noted<br>*Give P values as exact values whenever suitable.*
☐	☒	For Bayesian analysis, information on the choice of priors and Markov chain Monte Carlo settings
☐	☒	For hierarchical and complex designs, identification of the appropriate level for tests and full reporting of outcomes
☐	☒	Estimates of effect sizes (e.g. Cohen's *d*, Pearson's *r*), indicating how they were calculated

Our web collection on statistics for biologists contains articles on many of the points above.

Software and code

Policy information about availability of computer code

Data collection	The underlying code for this study is not publicly available but may be made available to qualified researchers on reasonable request from the corresponding author.
Data analysis	The underlying code for this study is not publicly available but may be made available to qualified researchers on reasonable request from the corresponding author.

For manuscripts utilizing custom algorithms or software that are central to the research but not yet described in published literature, software must be made available to editors and reviewers. We strongly encourage code deposition in a community repository (e.g. GitHub). See the Nature Portfolio guidelines for submitting code & software for further information.

Data

Policy information about availability of data

All manuscripts must include a data availability statement. This statement should provide the following information, where applicable:

- Accession codes, unique identifiers, or web links for publicly available datasets
- A description of any restrictions on data availability
- For clinical datasets or third party data, please ensure that the statement adheres to our policy

The source NHIS data do not belong to the researchers and we are not allowed to transfer data file to a third party under Korean law. The data can be used after approval of the Institutional Review Board and the Korea NHIS Big Data Operations Department (https://nhiss.nhis.or.kr/bd/ay/bdaya 001 iv.do).

Research involving human participants, their data, or biological material

Policy information about studies with human participants or human data. See also policy information about sex, gender (identity/presentation), and sexual orientation and race, ethnicity and racism.

Reporting on sex and gender	We investigated mental illnesses, including depression, bipolar disorder, schizophrenia, insomnia, and anxiety, which have been suggested to be related to PD, for their association with the risk of EOPD. We also analyzed the associations stratified by sex.
Reporting on race, ethnicity, or other socially relevant groupings	Lifestyle factors were assessed using a self-reported questionnaire. Alcohol consumption was classified according to the amount of alcohol consumed per day, and those with alcohol intake per day > 30 g were characterized as heavy drinkers. Regular physical activity was defined as engaging in ≥ 20 min of vigorous-intensity physical activity ≥ 3 days a week or ≥ 30 min of moderate-intensity physical activity ≥5 days a week. Individuals who paid the bottom 20% of NHI premiums were characterized to be in the low-income group. Anthropometric data, such as height and weight, were measured, and body mass index (BMI) was calculated as weight divided by height squared (kg/m2). Obesity was defined as BMI greater than 25. Laboratory measurements, such as fasting plasma glucose and total cholesterol levels, were conducted after overnight fasting. The baseline comorbidities of individuals were identified based on a combination of medical history information, ICD-10 codes, laboratory data, and prescribed medications.<br>Cox proportional hazards regression models were used to evaluate the risk of PD according to the presence of mental illness. We used four progressive models: Model 1 was unadjusted; Model 2 was adjusted for age and sex; Model 3 was further adjusted for smoking, alcohol consumption, and physical activity, income level, and BMI; and Model 4 was further adjusted for diabetes mellitus, hypertension, and dyslipidemia.
Population characteristics	Among participants aged <50 years, 330,726 (5.79%) were diagnosed with mental illness. Depression was diagnosed in 89,478 (27.06%), bipolar disorder in 6,645 (2.01%), schizophrenia in 10,030 (3.03%), insomnia in 84,293 (25.49%), and anxiety in 201,249 (60.8%). Mean age at the diagnosis of mental illness was 40.14 ± 7.19 years and men constituted 42.95%. People with mental illness were less likely to smoke or drink alcohol and more likely to perform regular physical activity than those without mental illness.<br>Among participants aged ≥50 years, 738,824 (17.54%) were diagnosed with mental illness. Depression was diagnosed in 230,648 (31.22%), bipolar disorder in 9,674 (1.31%), schizophrenia in 8,191 (1.11%), insomnia in 239,811 (32.46%), and anxiety in 458,452 (62.05%). Mean age at the diagnosis of mental illness was 63.08 ± 8.79 years and men constituted 33.99%. Table 1 displays the demographic and medical characteristics of participants stratified by age group.
Recruitment	We used data from the Korean National Health Insurance Service (NHIS) and National Health Screening databases for the analysis. The Korean NHIS is mandatory for Korean citizens with approximately 50 million subscribers. The database contains a unique anonymous number for each individual and includes demographic information as well as information related to medical records and costs, such as diagnoses using the International Classification of Diseases (ICD-10), examinations, prescriptions, and procedures. The NHIS provides a free national health screening program (NHSP) at least every 2 years for all beneficiaries aged ≥ 40 years and workplace subscribers of all ages. In addition to the physical measurements and laboratory tests, a self-reported questionnaire is administered.<br>Individuals aged ≥ 20 years, who had undergone a health examination provided by the NHIS in 2009, were enrolled. After excluding individuals with missing data, those who had a diagnosis of PD before enrollment were also excluded. We set a 1-year lag period for PD diagnosis to avoid the risk of reverse causality, and 9,920,544 individuals remained for the analysis. We categorized the remaining participants into two age-specific groups at enrollment and tracked them until December 31, 2018. Finally, 5,707,919 people aged < 50 years were enrolled, and participants were censored when they reached age of 50 years. A total of 4,212,063 individuals aged ≥50 years were included in the study. Among the participants, people with diagnoses of mental illness, including depression (F22, F23), bipolar disorder (F30, F31), schizophrenia (F20), insomnia (F510, G470), and anxiety (F40, F41), were identified based on ICD-10 codes (Figure 2).
Ethics oversight	The Institutional Review Board of the Korea University Guro Hospital

Note that full information on the approval of the study protocol must also be provided in the manuscript.

Field-specific reporting

Please select the one below that is the best fit for your research. If you are not sure, read the appropriate sections before making your selection.

☐ Life sciences ☒ Behavioural & social sciences ☐ Ecological, evolutionary & environmental sciences

For a reference copy of the document with all sections, see nature.com/documents/nr-reporting-summary-flat.pdf

Behavioural & social sciences study design

All studies must disclose on these points even when the disclosure is negative.

Study description	quantitative cohort study
Research sample	We used data from the Korean National Health Insurance Service (NHIS) and National Health Screening databases for the analysis. The Korean NsHIS is mandatory for Korean citizens with approximately 50 million subscribers. The database contains a unique

	anonymous number for each individual and includes demographic information as well as information related to medical records and costs, such as diagnoses using the International Classification of Diseases (ICD-10), examinations, prescriptions, and procedures. The NHIS provides a free national health screening program (NHSP) at least every 2 years for all beneficiaries aged ≥ 40 years and workplace subscribers of all ages. In addition to the physical measurements and laboratory tests, a self-reported questionnaire is administered.
Sampling strategy	Individuals aged ≥ 20 years, who had undergone a health examination provided by the NHIS in 2009, were enrolled. After excluding individuals with missing data, those who had a diagnosis of PD before enrollment were also excluded. We set a 1-year lag period for PD diagnosis to avoid the risk of reverse causality, and 9,920,544 individuals remained for the analysis. We categorized the remaining participants into two age-specific groups at enrollment and tracked them until December 31, 2018. Finally, 5,707,919 people aged < 50 years were enrolled, and participants were censored when they reached age of 50 years. A total of 4,212,063 individuals aged ≥50 years were included in the study. Among the participants, people with diagnoses of mental illness, including depression (F22, F23), bipolar disorder (F30, F31), schizophrenia (F20), insomnia (F510, G470), and anxiety (F40, F41), were identified based on ICD-10 codes (Figure 2).
Data collection	The Korean National Health Insurance Service (NHIS) and National Health Screening databases
Timing	Individuals aged ≥ 20 years, who had undergone a health examination provided by the NHIS in 2009, were enrolled. After excluding individuals with missing data, those who had a diagnosis of PD before enrollment were also excluded. We set a 1-year lag period for PD diagnosis to avoid the risk of reverse causality, and 9,920,544 individuals remained for the analysis. We categorized the remaining participants into two age-specific groups at enrollment and tracked them until December 31, 2018.
Data exclusions	Individuals aged ≥ 20 years, who had undergone a health examination provided by the NHIS in 2009, were enrolled. After excluding individuals with missing data, those who had a diagnosis of PD before enrollment were also excluded. We set a 1-year lag period for PD diagnosis to avoid the risk of reverse causality, and 9,920,544 individuals remained for the analysis. We categorized the remaining participants into two age-specific groups at enrollment and tracked them until December 31, 2018.
Non-participation	Not applicable
Randomization	Not applicable

Reporting for specific materials, systems and methods

We require information from authors about some types of materials, experimental systems and methods used in many studies. Here, indicate whether each material, system or method listed is relevant to your study. If you are not sure if a list item applies to your research, read the appropriate section before selecting a response.

Materials & experimental systems

n/a	Involved in the study
☒	☐ Antibodies
☒	☐ Eukaryotic cell lines
☒	☐ Palaeontology and archaeology
☒	☐ Animals and other organisms
☒	☐ Clinical data
☒	☐ Dual use research of concern
☒	☐ Plants

Methods

n/a	Involved in the study
☒	☐ ChIP-seq
☒	☐ Flow cytometry
☒	☐ MRI-based neuroimaging

Plants

Seed stocks	*Report on the source of all seed stocks or other plant material used. If applicable, state the seed stock centre and catalogue number. If plant specimens were collected from the field, describe the collection location, date and sampling procedures.*
Novel plant genotypes	*Describe the methods by which all novel plant genotypes were produced. This includes those generated by transgenic approaches, gene editing, chemical/radiation-based mutagenesis and hybridization. For transgenic lines, describe the transformation method, the number of independent lines analyzed and the generation upon which experiments were performed. For gene-edited lines, describe the editor used, the endogenous sequence targeted for editing, the targeting guide RNA sequence (if applicable) and how the editor was applied.*
Authentication	*Describe any authentication procedures for each seed stock used or novel genotype generated. Describe any experiments used to assess the effect of a mutation and, where applicable, how potential secondary effects (e.g. second site T-DNA insertions, mosiacism, off-target gene editing) were examined.*
